# A Pan-Pneumovirus vaccine based on immunodominant epitopes of the fusion protein

**DOI:** 10.3389/fimmu.2022.941865

**Published:** 2022-08-08

**Authors:** Jiachen Huang, Rose J. Miller, Jarrod J. Mousa

**Affiliations:** ^1^Department of Infectious Diseases, College of Veterinary Medicine, University of Georgia, Athens, GA, United States; ^2^Center for Vaccines and Immunology, College of Veterinary Medicine, University of Georgia, Athens, GA, United States; ^3^Department of Biochemistry and Molecular Biology, Franklin College of Arts and Sciences, University of Georgia, Athens, GA, United States

**Keywords:** RSV (respiratory syncytial virus), human metapneumovirus (hMPV), vaccine, structure-based vaccine design, neutralizing antibodies, fusion protein, epitope

## Abstract

Respiratory syncytial virus (RSV) and human metapneumovirus (hMPV) are two leading causes of severe respiratory infections in children, the elderly, and immunocompromised patients. The fusion (F) protein is the major target of neutralizing antibodies. Recent developments in stabilizing the pre-fusion conformation of the F proteins, and identifying immunodominant epitopes that elicit potent neutralizing antibodies have led to the testing of numerous pre-fusion RSV F-based vaccines in clinical trials. We designed and tested the immunogenicity and protective efficacy of a chimeric fusion protein that contains immunodominant epitopes of RSV F and hMPV F (RHMS-1). RHMS-1 has several advantages over vaccination with pre-fusion RSV F or hMPV F, including a focus on recalling B cells to the most important protective epitopes and the ability to induce protection against two viruses with a single antigen. RHMS-1 was generated as a trimeric recombinant protein, and analysis by negative-stain electron microscopy demonstrated the protein resembles the pre-fusion conformation. Probing of RHMS-1 antigenicity using a panel of RSV and hMPV F-specific monoclonal antibodies (mAbs) revealed the protein retains features of both viruses, including the pre-fusion site Ø epitope of RSV F. Mice immunized with RHMS-1 generated neutralizing antibodies to both viruses and were completely protected from RSV or hMPV challenge. Overall, this study demonstrates protection against two viruses with a single antigen and supports testing of RHMS-1 in additional pre-clinical animal models.

## Introduction

Respiratory syncytial virus (RSV) and human metapneumovirus (hMPV) are significant causes of acute lower respiratory tract infections (ALRI) in infants and young children (1–4). RSV was first identified in 1956, and was subsequently recognized as a common cause of respiratory illness in early life (5). The majority of children experience at least one RSV infection before 2 years of age, and infants under 6 months old have a higher risk of severe disease requiring hospitalization (6). hMPV was identified in 2001, and it is the second most common cause of viral lower respiratory infection in children (7). In contrast to RSV, the peak age for infant hospitalizations caused by hMPV infections is 6-12 months old, with a nearly 100% exposure rate by the age of 5 (3). Reinfections of both RSV and hMPV are common throughout life, which usually cause mild symptoms in healthy adults. However, for certain populations including immunocompromised patients, individuals over 65 years of age, and people with underlying conditions such as asthma or chronic obstructive pulmonary disease (COPD), infection with RSV or hMPV may lead to severe bronchiolitis and pneumonia (8–11).

Both hMPV and RSV are enveloped negative-sense single-stranded RNA viruses that belong to the Pneumoviridae family (12). The 15.2 kb genome of RSV consists of 10 genes (encoding 11 proteins), while the 13.3 kb hMPV genome has 8 open reading frames (lacking NS1 and NS2). On the membrane of the Pneumovirus virion, the attachment (G) protein is responsible for viral adherence, the fusion (F) protein mediates membrane fusion and viral entry to host cells (13), while the small hydrophobic (SH) protein is a putative viroporin that forms ion channels and is involved in the prevention of apoptosis of infected cells (14, 15). RSV F and G as well as hMPV F proteins are the main targets of neutralizing antibodies. However, SH is poorly immunogenic and cannot induce neutralizing antibodies, likely due to its small size and low abundance on the virions (16). The M2 gene of RSV and hMPV encodes two overlapping open reading frames for M2-1 and M2-2 proteins. The viral capsid is made from a layer of the matrix (M) and M2-1 proteins (17) where M2-1 connects M with the internal ribonucleoprotein complexes (RNPs) of viral genomic RNA, nucleoprotein (N), large RNA polymerase (L), and the phosphoprotein (P). M2-2 is served as a regulator that is involved in the balance between RNA synthesis and transcription in both RSV and hMPV (18, 19). The RSV non-structural proteins NS1 and NS2 are involved in the inhibition of alpha/beta interferons (IFN-α/β) *in vitro* (20, 21), and RSV infection was attenuated without NS1 and NS2 (22, 23).

The F glycoproteins of RSV and hMPV are highly similar in structure and share ~30% amino acid sequence identity. Both F proteins belong to the class I viral fusion protein family and play indispensable roles in viral attachment as well as membrane fusion. To become fusion competent, the F0 precursor must be cleaved into F1 and F2 subunits that are linked by two disulfide bonds to generate a mature meta-stable homotrimer (24). RSV F is cleaved at two furin cleavage sites with the p27 fragment in between F1 and F2 removed, whereas hMPV F has only one cleavage site that can be cleaved by the host membrane protease TMPRSS2 (25). To initiate the fusion process, the hydrophobic fusion peptide on the N terminus of the F2 subunit is exposed and inserted into the host cell membrane, which triggers the conformational rearrangements that turn the F protein into the stable post-fusion state, and brings the viral and host cell membranes together for lipid mixing.

Multiple antigenic sites have been identified on both RSV F and hMPV F proteins. Among the six known antigenic sites of RSV F, pre-fusion-specific sites Ø and V are targeted by over 60% of neutralizing antibodies in humans (26, 27), indicating these sites are vital for immune recognition and antibody neutralization (28). hMPV F shares three antigenic sites (III, IV, V) with RSV F, as several antibodies have been found to cross-react with RSV and hMPV F at these epitopes (29–32). In addition, the area between sites III and IV was found to be a distinct hMPV site that is recognized by a mAb called DS7 (33, 34). Studies have shown that, unlike RSV F-specific antibodies, the majority of hMPV F-specific antibodies target epitopes present in both pre-fusion and post-fusion conformations, likely due to glycosylation present near pre-fusion-specific sites on the head of hMPV F (35, 36).

Currently, there are no vaccines available for either RSV or hMPV. Previous attempts with formalin-inactivated RSV and hMPV vaccines revealed that low affinity, non-neutralizing F-specific antibodies induced by denatured post-fusion proteins cannot provide protection, and lead to vaccine-enhanced disease (37–41). By stabilizing the RSV F protein in the pre-fusion conformation, several studies have demonstrated improvement in neutralizing antibody titers (42, 43). The bivalent Pfizer RSV vaccine that contains pre-fusion F from RSV subgroups A and B (44) and the pre-fusion RSV F vaccine from GlaxoSmithKline (GSK) (45) are two leading candidates that under phase III clinical trials at present. For hMPV, pre-fusion and post-fusion F proteins induced comparable neutralizing antibodies in mice (36) and immunization with post-fusion F completely protected mice from hMPV challenge (46). Several epitope-focused vaccine designs have been tested for RSV and hMPV F. A head-only RSV F protein boosted titers of neutralizing Abs targeting antigenic sites Ø and II (47). In a different study, RSV F was modified by glycan-masking that blocked poorly neutralizing epitopes on a nanoparticle, which induced a more potent neutralizing Ab response than a pre-fusion F trimer (48). Based on computational protein design strategies, RSV F site II was presented on a scaffold fused with RSV N-based nanoparticles, which boosted subdominant neutralizing antibody responses targeting antigenic site II in mice (49, 50). In addition, RSV F neutralizing antigenic sites (Ø, II, IV) were tested on *de novo* protein scaffolds respectively, and a mixture of these epitope-based immunogens induced focused immune responses toward the target antigenic sites (51). All of the studies above demonstrate the concept that engineered RSV F epitope-based immunogens can induce and boost neutralizing RSV F antibodies.

The idea of universal vaccine development provides the possibility of preventing multiple viruses/viral variants by a single immunogen. Due to the similarities between RSV and hMPV F, researchers have tried to generate universal RSV/MPV vaccines by grafting the helix-turn-helix motif (site II) from RSV F onto hMPV F, however, this chimera induced neutralizing antibody responses only to hMPV, but not RSV (52). A similar study that grafted RSV F and hMPV F epitopes on pre-fusion and post-fusion F proteins showed that chimeric proteins swapping either site II or site IV can induce cross-neutralizing antibodies in mice, but a challenge with pre-fusion candidates was lacking (53). For the influenza hemagglutinin (HA) protein, chimeric immunogens generated by swapping the HA head with zoonotic subtypes while retaining the conserved HA stem successfully induced antibodies targeting the subdominant HA stem (54, 55).

Based on these findings and the knowledge about structures and the immunodominant epitopes of RSV and hMPV F proteins, we designed a novel chimeric immunogen that contains the head of RSV F and the stem of hMPV F. The **R**SV **h**ead h**M**PV **s**tem construct **1** (RHMS-1) protein was stably expressed as a pre-fusion trimer that preserved the structural features on key antigenic sites for both RSV and hMPV F proteins. RHMS-1 retains immunodominant epitopes of both F proteins, including antigenic sites Ø, V, and II of RSV F, and sites IV, DS7, and III of hMPV F. Immunization of mice with RHMS-1 induced potent neutralizing antibodies that protected mice from both RSV and hMPV challenge. Overall, our data demonstrate that RHMS-1 can be a promising universal vaccine against both Pneumoviruses.

## Materials and methods

### Expression and purification of proteins

Plasmids encoding cDNAs of Pneumovirus fusion proteins were synthesized (GenScript) and cloned into the pcDNA3.1^+^ vector. The stable cell line that expresses the hMPV B2 F protein was utilized as previously described (46, 56). The rest of the F proteins and monoclonal antibodies (mAbs) were transiently expressed in Expi293F cells or from hybridomas (for mAbs) as previously described (56). The proteins were harvested from the supernatant of cell cultures and purified by HisTrap Excel (for his-tagged proteins) or Protein G (for antibodies) columns (GE Healthcare Life Sciences). RHMS-1 (trimer), RSV A2 F DsCav1 (trimer) (42), and trypsin-treated hMPV B2 F (monomer) were further purified by size exclusion chromatography on a Superdex S200, 16/600 column (GE Healthcare Life Sciences). The influenza A/California/07/2009 hemagglutinin (HA) protein was expressed and purified as previously described (57).

### Negative-stain electron microscopy analysis

Purified RHMS-1 (trimer) was applied on carbon-coated copper grids (5 μL of 10 μg/mL protein solution) for 3 min. The grid was washed in water twice and then stained with Nano-W (Nanoprobes) for 1 min. Negative-stain electron micrographs were acquired using a JEOL JEM1011 transmission electron microscope equipped with a high-contrast 2K-by-2K AMT midmount digital camera.

### ELISA of RHMS-1 with mAbs or human/mouse serum

384-well plates (Greiner Bio-One) were coated with 2 μg/mL of antigen in PBS overnight at 4°C. The plates were then washed once with water before blocking for 1 hour with the blocking buffer. Primary mAbs (starting at 20 μg/mL and followed by 3-fold dilutions) or serial dilutions of human/mouse serum (starting with 1:50 and followed by 3-fold dilutions) were applied to wells for 1 hour after three washes with water. Plates were washed with water three times before applying 25 μL of secondary antibody (goat anti-human IgG Fc-AP Southern Biotech, 2048-04; goat anti-mouse IgG Fc-AP Southern Biotech, 1033-04) at a dilution of 1:4,000 in blocking buffer. After incubation for 1 hour, the plates were washed five times with 0.05% PBS-Tween-20, and 25 μL of a PNPP (p-nitrophenyl phosphate) substrate solution (1 mg/mL PNPP in 1 M Tris base) was added to each well. The plates were incubated at room temperature for 1 hour before reading the optical density at 405 nm (OD_405_) on a BioTek plate reader. Data were analyzed in GraphPad Prism using a nonlinear regression curve fit and the log(agonist)-versus-response function to calculate the binding EC_50_ values. Mouse serum IgG endpoint titers were calculated from the highest dilution of a serum sample that produced OD_405_ readings of >0.3 above the background readings and were shown in a log_10_ scale as previously described (46).

### Binding kinetics of fusion proteins with human mAbs by biolayer interferometry

SEC Purified hMPV F 130BV, RSV F DsCav1, and RHMS-1 were loaded onto HIS1K biosensors at 100 μg/mL for 60 s, then the binding kinetics of mAbs (10 μg/mL) were analyzed by association for 300 s and dissociation for 300 s. Octet data analysis software was used to analyze the data. Binding curves were independently graphed in GraphPad Prism for data visualization.

### ELISA screening of human PBMCs

As previously described (58), peripheral blood mononuclear cells (PBMCs) and plasma were isolated from human subject blood samples using CPT tubes (BD, 362753), and PBMCs were frozen in the liquid nitrogen vapor phase until further use. For serology screening, the plasma samples of 41 subjects were used for ELISA as described above. The IgG binding was quantified by the area under the curve (AUC) values using GraphPad Prism. For PBMC screening, 4 of the 41 subjects were selected, and for each subject, ten million PBMCs were mixed with 8 million previously frozen and gamma-irradiated NIH 3T3 cells modified to express human CD40L, human interleukin-21 (IL-21), and human B-cell activating factor (BAFF) in 80 mL StemCell medium A (StemCell Technologies) containing 1 μg/mL of cyclosporine A (Millipore-Sigma). The mixture of cells was plated in four 96-well plates at 200 μL per well in StemCell medium A. After 6 days, undiluted culture supernatants were screened by ELISA for IgG binding to the RHMS-1 (trimer), RSV A2 DsCav1 F (trimer), and trypsin-treated hMPV B2 F (monomer). Each well is represented by a dot with the OD_405 nm_ against RSV/hMPV F as the x coordinate, and the OD_405 nm_ against RHMS-1 as the y coordinate.

### Animal immunization and hMPV/RSV challenge

BALB/c mice (6 to 8 weeks old; The Jackson Laboratory) were immunized in a prime-boost regimen with 25 μL purified RHMS-1 (trimer), RSV A2 DsCav1 F (trimer), or trypsin-treated hMPV B2 F (monomer) (20 μg protein/mouse) + 25 μL AddaS03 adjuvant *via* the subcutaneous route into the loose skin over the neck, while mice in control groups were immunized with PBS + AddaS03 adjuvant (8 animals per group). Three weeks after prime, the mice were boosted with the same amount of the antigens + adjuvant. Three weeks after the boost, mice were bled and then intranasally challenged with RSV A2 (2.8x10^6^ PFU per mouse) or hMPV TN/93-32 (3x10^5^ PFU per mouse). Mice were sacrificed 5 days post-challenge, and lungs were collected and homogenized for virus titration as previously described (46). Briefly, RSV-challenged lung homogenates were plated on HEp-2 cells (EMEM+2% FBS) while hMPV-challenged lung homogenates were plated on LLC-MK2 cells (EMEM + 5 μg/mL trypsin-EDTA and 100 μg/mL CaCl_2_) in 24 well plates. After 4-5 days, the cells were fixed with 10% neutral buffered formalin and the plaques of both viruses were immunostained with human mAbs MPV364 (for hMPV) (58) or 101F (for RSV) (59). Plaques were counted under a stereomicroscope.

### Virus neutralization assays with immunized mice sera

For serum neutralization assays, the serum of 4/8 mice were randomly picked from each group. Heat-inactivated mouse serum was serially diluted (starting at 1:25 and followed by 3-fold dilutions) and incubated 1:1 with a suspension of hMPV (CAN/97-83 and TN/93-32) or RSV (A2 and B) for 1 hour at room temperature. PBS or serially diluted serum samples from naïve mice were mixed with viruses as the negative control. LLC-MK2 (for hMPV) or HEp-2 cells (for RSV) in 24-well plates were then inoculated with the serum-virus mixture (50 μL/well) for 1 hour and rocked at room temperature before adding the overlay (0.75% Methylcellulose in EMEM+ 2% FBS for HEp-2 cells; 0.75% Methylcellulose in EMEM + 5 μg/mL trypsin-EDTA and 100 μg/mL CaCl_2_ for LLC-MK2 cells). After 4-5 days, the plaques were stained as described above. The percent neutralization was calculated by (PFU in control wells − PFU in serum wells)/PFU in control wells × 100%.

### Competition binding of human mAbs with immunized mice sera by biolayer interferometry

RSV A2 DsCav1 and hMPV F 130BV, as well as all the human reference mAbs were diluted at 100 μg/mL in Octet buffer (PBS + 0.02% Tween20, 0.1% BSA) and loaded onto HIS1K Biosensors. Vaccinated mice serum samples from each vaccination group were pooled and serial diluted (1/10, 1/100, and 1/1000) in ChonBlock blocking buffer (25% ChonBlock buffer diluted in Octet buffer) (Chondrex Inc.), while pooled serum from PBS vaccinated mice was diluted at 1/10 in Chonblock blocking buffer. The loaded biosensors were immersed into wells containing diluted serum samples for 300 s, the non-specific binding signal was then removed by immersing the sensors in Octet buffer. Following this, biosensors were immersed into wells containing 100 µg/mL of a human reference mAb that binds to different epitopes for 300 s. Percent inhibition of pooled mice serum was calculated by (1 – max signal of the vaccinated serum/max signal of the naive serum) × 100%.

### Analysis of mouse IgG isotypes in mouse serum

IgG isotype responses in mouse serum were measured by ELISA. High-binding 384 well plates were coated with antigens corresponding to immunization groups (RHMS-1, RSV A2 DsCav1, hMPV B2 F monomer) as described above. After blocking and washing the plate, serially diluted serum samples (pooled together for each mouse group) in blocking buffer (1:100, followed by 3-fold dilutions) were added to the plate. After incubation for 1 hr, the plates were washed three times with water. Each IgG isotype secondary antibody (goat anti-mouse IgG_1_ Fc-AP Southern Biotech, 1070-04; goat anti-mouse IgG_2a_ Fc-AP Southern Biotech, 1080-04; goat anti-mouse IgG_2b_ Fc-AP Southern Biotech, 1090-04; goat anti-mouse IgG_2c_ Fc-AP Southern Biotech, 1079-04; goat anti-mouse IgG_3_ Fc-AP Southern Biotech, 1100-04) was diluted 1:4,000 in blocking buffer. Following incubation, the plate was developed with the PNPP substrate solution, the OD_405_ values were measured, and the IgG endpoint titers were determined and graphed on GraphPad Prism as described above.

### Th1/Th2 cytokine analysis of mouse lungs

Lung homogenates were thawed on ice and centrifuged at 10,000 x g for 10 minutes. The supernatant (pooled together for each mouse group) was used for measuring IFN-γ, IL-2, IL-4, and IL-10 levels by using a mouse Th1/Th2 ELISA kit (ThermoFisher, 88–7711-44) per the manufacturer’s instructions. Undiluted samples were normalized to the baseline OD_405_ values and the concentrations of each cytokine were calculated based on each respective standard curve.

## Results

### RHMS-1 was expressed as a pre-fusion trimer

**Figure 1:**
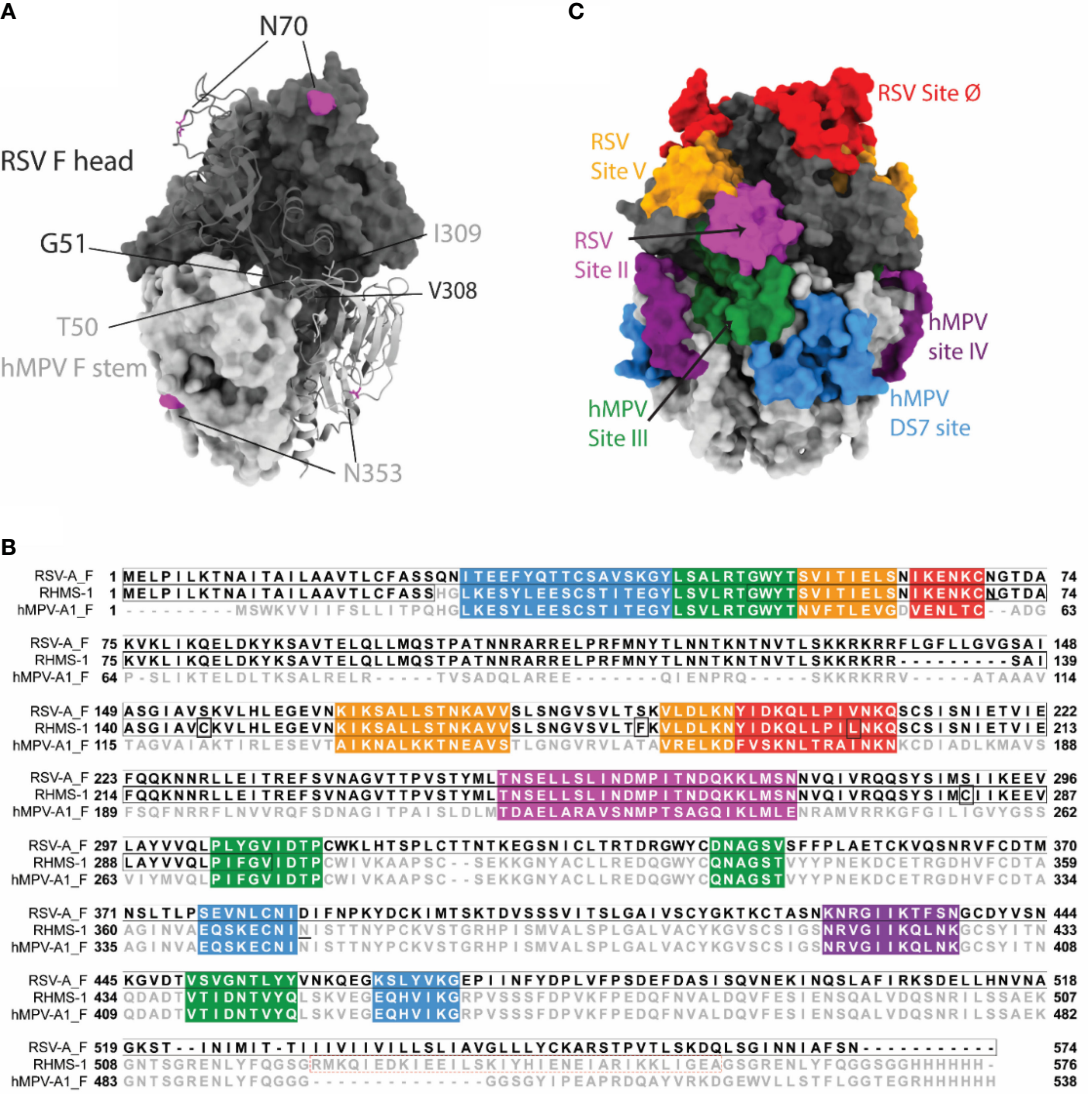
RHMS-1 protein design. **(A)** The diagram generated with the head (gray) of pre-fusion RSV F (5UDE) and the stem (silver) of pre-fusion hMPV F (5WB0) shows one protomer in cartoon and the rest of two protomers in surface. The glycosylation sites are shown in magenta. **(B)** Sequence alignment of RSV-A F, RHMS-1, and hMPV-A1 F generated by Jalview. The sequences of known antigenic sites are highlighted: RSV site Ø – red, RSV site V – orange, RSV site II – magenta, hMPV site III – forest, hMPV site IV – purple, hMPV DS7 site – blue. In RHMS-1 sequence, four Ds-Cav1 mutations are circled in boxes, two N-linked glycosylation sites are underlined and the GCN4 trimerization domain is circled in red dashed box. **(C)** The antigenic sites colored in accordance with the sequences highlighted in Figure 1B are displayed on the diagram. Both figure 1A and C were made by ChimeraX.

We designed the RHMS-1 protein based on the pre-fusion structures of RSV F (5UDE) and hMPV F (5WB0) using ChimeraX (60), and a model of RHMS-1 based on these structures is shown in **Figure 1A**. The design of RHMS-1 maintains the signal peptide, two cleavage sites, DsCav1 mutations (S115C, S190F, V207L, S290C) (42), and the fusion peptide of RSV F. Part of the F2 N-terminus (residues 26-54) and the F1 C-terminus (residues 315-531) was replaced by the homologous hMPV F sequences, with two junctions located on β2 and β7 strands. Two glycosylation sites (RSV F-N70, hMPV F-N353) are retained in RHMS-1. A GCN4 trimerization domain and a hexa-histidine tag were appended to the F1 C-terminus (**Figure 1B**). Intact RSV F sites II, V, and Ø and hMPV F sites IV, site III, and DS7 site were adopted from the original sequence (**Figure 1C**).

**Figure 2:**
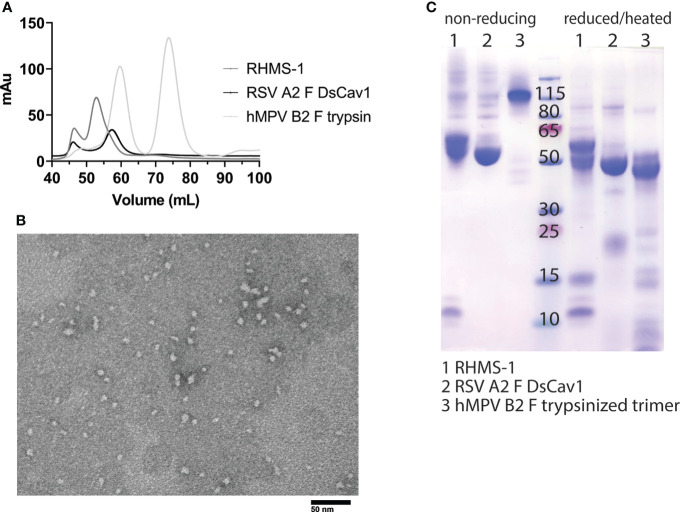
Purification and Negative-stain EM of RHMS-1. **(A)** Size exclusion chromatography curves of RHMS-1 (gray), RSV A2 F DsCav1 (black), and trypsinized hMPV B2 F (silver). mAu: milli absorbance units. **(B)** SDS-PAGE of F proteins in non-reducing and reduced/heated conditions. Numbers shown at the marker lane represent kDa values. **(C)** Representative negative-stain electron micrograph of RHMS-1 obtained from fractions 50-60 mL from the size exclusion chromatogram shown in **(A)**.

RHMS-1 was expressed in HEK293F cells and size exclusion chromatography (SEC) showed RHMS-1 was mainly expressed as a trimeric protein, but the size was slightly bigger than RSV F trimers (**Figure 2A**). For hMPV F, trimers and monomers were observed after trypsin treatment as previously described (46), but the size of trimeric hMPV F is smaller than RHMS-1 and RSV F, likely due to trypsinization (**Figure 2A**). Like RSV F, RHMS-1 was deduced to be cleaved after expression, as the F2 domain (~15 kDa) was observed on the gel under reducing conditions (**Figure 2B**), and the sizes of RHMS-1 and RSV F bands are consistent with the peaks shown in **Figure 2A**. Negative-stain EM analysis demonstrated that the majority of RHMS-1 particles were in the pre-fusion conformation based on “ball-like” structures resembling pre-fusion RSV and hMPV F (36, 42) (**Figure 2C**), indicating the DsCav1 mutations work well in stabilizing the structure of RHMS-1.

### RHMS-1 shares immunological features of both RSV and hMPV F

**Figure 3:**
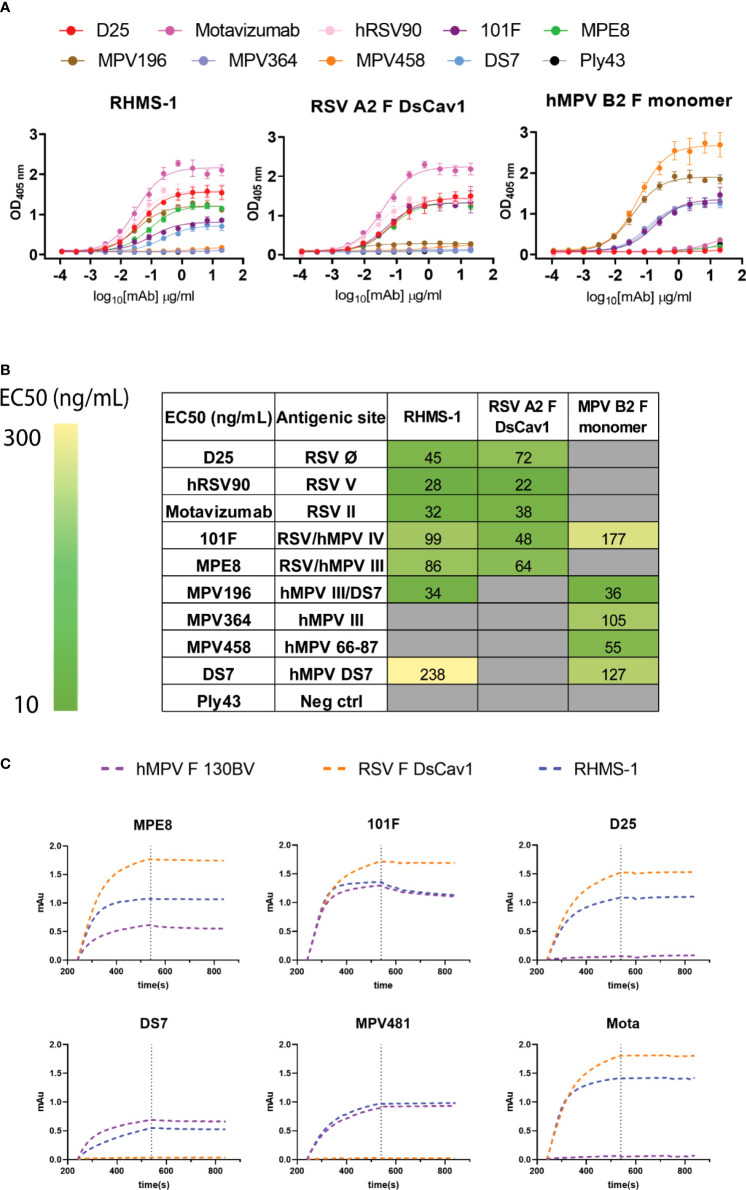
Antigenic site-specific mAbs binding to F proteins. **(A)** ELISA binding curves of mAbs targeting different RSV/hMPV F antigenic sites against RHMS-1, RSV A2 F DsCav1, and trypsinized hMPV B2 F monomer. **(B)** EC_50_ values of the binding curves in **(A)**. The binding curves and the EC_50_ values were generated by GraphPad Prism. **(C)** BLI binding kinetics of RHMS-1, RSV A2 F DsCav1, and hMPV F 130BV with reference mAbs.

To determine if RHMS-1 retains the correct conformation of each antigenic site, mAbs specifically targeting these sites were tested for binding by ELISA (**Figure 3A**). For both RSV F and RHMS-1, mAb D25 binds to site Ø (61), mAb hRSV90 binds to site V (62), and motavizumab binds to site II (63, 64) at similar EC_50_ values (**Figure 3B**). For both hMPV F and RHMS-1, mAbs DS7 and MPV196 bind to the DS7 site (33, 58). mAb 101F binds to all three antigens on site IV (29) while mAb MPE8 binds to site III on RSV F and RHMS-1 (65), but not monomeric hMPV F, likely due to the cross-protomer epitope that is only partially displayed on hMPV F monomer. The binding site of mAb MPV364 partially overlaps with hMPV site III, but it was also predicted to interact with the head of hMPV F (58), and mAb MPV458 binds to the 66-87 peptide on the head of hMPV F (56), therefore, both mAbs MPV364 and MPV458 do not bind to RHMS-1 as expected. In addition, we determined the binding kinetics of trimeric hMPV F, RSV F, and RHMS-1 against reference mAbs through BLI (**Figure 3C**). In consistence with the ELISA data, mAbs MPE8 and 101F bind to all three constructs. mAbs D25 and motavizumab showed similar binding kinetics with RSV F and RHMS-1, while mAbs DS7 and a hMPV F-specific site IV mAb, MPV481 (66), showed similar binding kinetics with hMPV F and RHMS-1. Overall, our data suggest that RHMS-1 maintains the conformational structures on the included antigenic epitopes from the RSV F and hMPV F proteins.

### RHMS-1 can be recognized by B cells pre-exposed to RSV F or hMPV F

**Figure 4:**
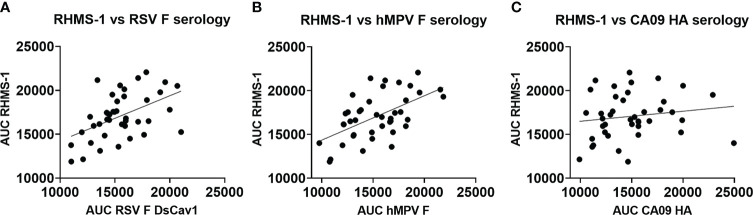
Serology of human plasma against F proteins. Area under the curve analysis of plasma IgG binding to RHMS-1 vs. RSV A2 F DsCav1 **(A)**, RHMS-1 vs. trypsinized hMPV B2 F monomer **(B)**, and RHMS-1 vs. influenza HA (A/California/07/2009). Each dot represents one subject, and the lines indicate the linear regression fit of the data sets (R^2^ = 0.174, p=0.0067 for **(A)**; R^2^ = 0.324, p=0.0001 for **(B)**; R^2^ = 0.023, p=0.347 for **(C)**]. Figures and data analysis was generated by GraphPad Prism.

**Figure 5:**
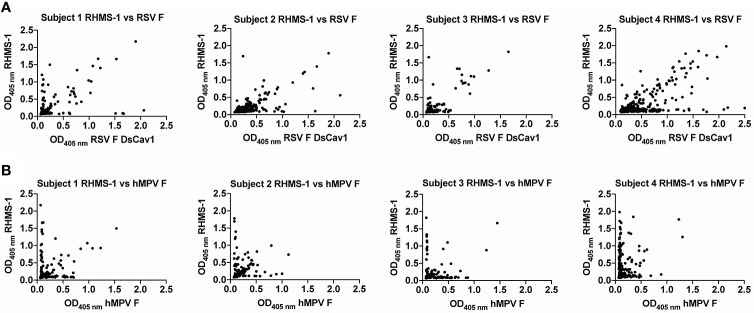
Human PBMCs binding to F proteins. ELISA OD_405 nm_ values of B cell culture supernatants binding to RHMS-1 vs. RSV A2 F DsCav1 **(A)** and RHMS-1 vs. trypsinized hMPV B2 F monomer **(B)**. Each dot represents the B cell supernatant in a single well of a 384 well plate initially containing 20,000 PBMCs. Figures were generated by GraphPad Prism.

To verify if epitopes on RHMS-1 can be recognized by the human immune system in a similar manner compared to RSV F or hMPV F, we screened plasma IgG responses from 41 human subjects against these proteins by ELISA. Overall, a simple linear regression fit showed positive correlations of serum IgG bindings for both RSV F vs. RHMS-1 (**Figure 4A**) and hMPV F vs. RHMS-1 (**Figure 4B**), but no correlation was observed for an irrelevant antigen, influenza A/California/07/2009 HA protein vs. RHMS-1 (**Figure 4C**). The serological screening data suggest epitopes are conserved between the native F proteins and the epitopes included on RHMS-1. The antibody responses at the cellular level were also tested by measuring the binding of supernatant from stimulated B cells in four subjects. The B cells in PBMCs were activated through coincubation with NIH 3T3 cells expressed human CD40L, human interleukin-21 (IL-21), and human B-cell activating factor (BAFF) to stimulate growth and IgG secretion to the culture supernatant as previously described (58). For all of the subjects we tested, the majority of RSV F-positive B cells are also positive for RHMS-1 (**Figure 5A**). This finding is expected as the majority of human B cells target the head of the RSV F protein, which is retained in RHMS-1. Such correlations are still present for hMPV F (**Figure 5B**), although the frequencies of hMPV F-positive B cells are generally lower than RSV F-positive B cells, therefore, populations of hMPV F negative, RHMS-1 positive B cells close to the Y axes were seen.

### Vaccination with RHMS-1 elicits potent neutralizing antibodies and cross-protection in mice

**Figure 6:**
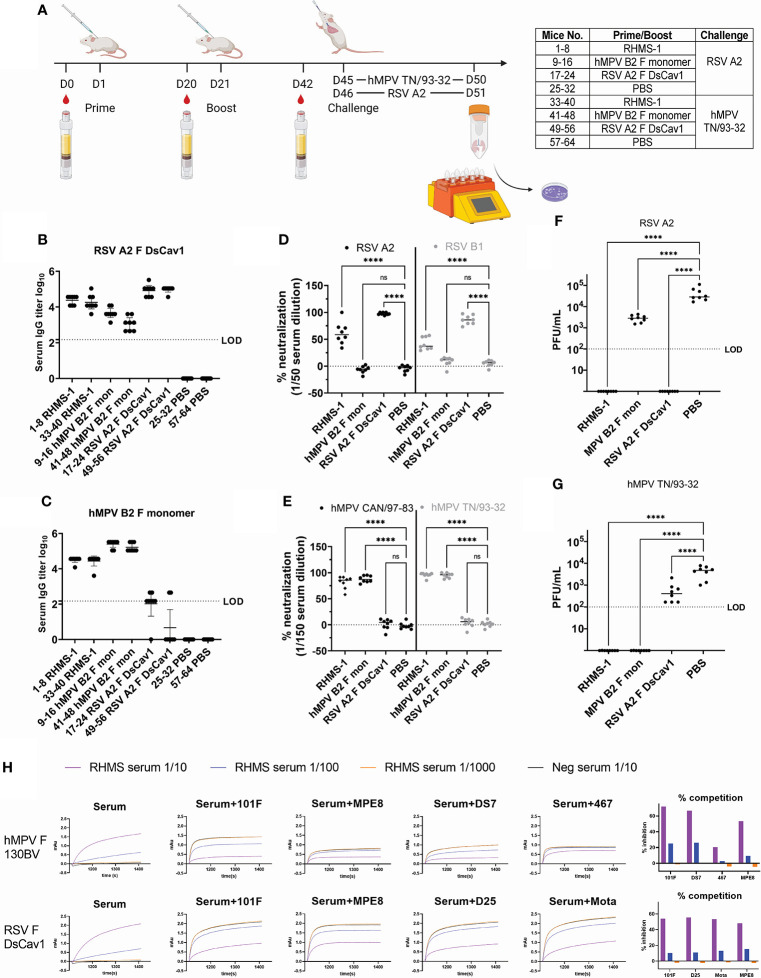
Mouse immunization and challenge studies. **(A)** Study regimen. Day 42 serum IgG titers against RSV A2 F DsCav1 **(B)** and trypsinized hMPV B2 F monomer **(C)** before challenge. **(D)** Serum neutralization against RSV A2 and B1 strains at 1/50 dilution and against **(E)** hMPV CAN/97-83 and TN/93-32 at a 1/150 dilution. Viral titers for RSV A2 **(F)** and hMPV TN/93-32 **(G)** in the lung homogenates of mice 5 days post-challenge. LOD, limit of detection. **(H)**. Competition binding of RHMS-1 (serial diluted at 1/10, 1/100, and 1/1000) or mock (1/10 diluted) vaccinated Day 42 serum with reference mAbs. ns, not significant. ****P < 0.0001.

To evaluate the immunological properties of RHMS-1, it was tested as a vaccine in the mouse model. BALB/c mice were subcutaneously primed and boosted with 20 μg of RHMS-1, RSV F DsCav1, hMPV monomeric B2 F, or PBS in an emulsion formulated with AddaS03 adjuvant and then challenged with RSV or hMPV (**Figure 6A**). All RHMS-1 vaccinated mice showed serum IgG binding titers against both RSV F DsCav1 and hMPV monomeric B2 F proteins (**Figure 6B, C**). Representative viruses from each RSV subgroup and each hMPV genotype were neutralized by RHMS-1 immunized mouse serum 3 weeks after the boost (**Figure 6D, E**). RSV F DsCav1 immunization failed to induce IgG that cross-recognize hMPV monomeric B2 F, while hMPV monomeric B2 F immunized mice showed moderate binding, but non-neutralizing IgG against RSV. Three weeks after the boost, mice were intranasally challenged with RSV A2 or hMPV TN/93-32. The virus titers in the lung homogenate were determined 5 days post challenge. Vaccination with RHMS-1 completely protected the mice from the challenges of both viruses, while RSV A2 F DsCav1 and hMPV B2 F monomer vaccinated groups protected mice only against the autologous virus (**Figure 6F, G**).

To determine if RHMS-1 induced epitope-specific antibodies against RSV F head and hMPV F stem, pooled Day 42 serum samples were used for competition binding assay by BLI (**Figure 6H**). For hMPV F 130BV, RHMS-1 vaccinated serum showed potent competition with mAbs 101F, MPE8, and DS7, but not with the hMPV F head-specific antibody mAb MPV467 (66). For RSV F DsCav1, RHMS-1 vaccinated serum (1/10 diluted) inhibited the binding of mAbs MPE8, 101F, D25, and motavizumab by ~50% compared to the mock vaccinated serum. Interestingly, RHMS-1 induced site III and site IV-competing antibodies for both RSV F and hMPV F, which may be due to the similar sequences at these 2 sites (**Figure 1B**). Another potential explanation is that site III and site IV are located at the interface of RHMS-1, and certain binding angles of antibodies on the RSV F head may interfere with the antibodies that bind to the hMPV F stem or vice versa.

### No sign of vaccine-enhanced disease was observed based on IgG isotype and cytokine profiles

**Figure 7:**
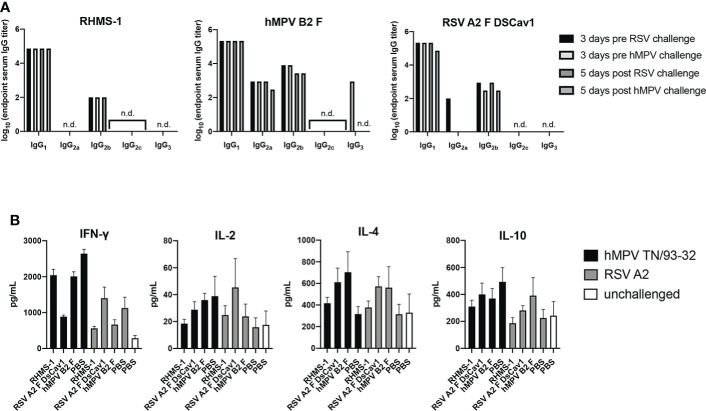
TH1/TH2 antibody and cytokine response after immunization and challenge. **(A)** Mouse serum IgG isotype ELISA against each vaccinating antigen (RHMS-1, hMPV B2 F, RSV A2 DsCav1) three days prior to viral challenge and five days post-challenge (hMPV TN/93-32, RSV A2). n.d., no detection above OD405 = 0.3. **(B)** Cytokine ELISA in the lung homogenates of mice five days post-challenge (hMPV TN/93-32, RSV A2). Error bars are the standard deviation.

To further assess the immune response elicited by vaccination and challenge, we determined the IgG isotypes elicited by immunization in mice (**Figure 7A**). Serum for each vaccine group was measured for binding against the vaccinating antigen, and we observed RHMS-1 vaccinated mice had a predominantly IgG_1_ immune response, suggestive of a Th2-skewed immune response (67). Similar results were obtained for hMPV B2 F and RSV A2 F DsCav1 immunized mice with additional IgG_2a_ antibody detected for these groups. BALB/c mice have a Th2-skewed immune response (68), and these data indicate the AddaS03 adjuvant cannot overcome this bias for these antigens. We further assessed the in mouse lungs cytokine response after RSV and hMPV challenge (**Figure 7B**). IFN-γ, IL-2, IL-4, and IL-10 responses were measured and compared to control vehicle immunized and challenged mice, as well as mice that were not immunized or challenged. We observed increases in IFN-γ responses for all challenged groups. IL-4 responses, indicating a Th2 immune response, were highest in the RSV A2 F and hMPV B2 F immunized mice compared to RHMS-1, suggesting RHMS-1 can limit the Th2 immune responses associated with potential vaccine enhanced disease.

## Discussion

Multiple protein engineering strategies have been investigated to generate cross-protective or epitope-based antigens against RSV F or hMPV F. Previous attempts at grafting a single antigenic site on RSV F or hMPV F from one to another induced cross-neutralizing antibodies but very limited protection against the heterologous virus challenge, likely due to the epitopes on the backbone still dominate the immune responses (53). RHMS-1 contains multiple immunodominant epitopes of both RSV F and hMPV F in relatively equal proportions, including at least three RSV F-specific and three hMPV F-specific antigenic sites. Therefore, more balanced immune responses against both RSV F and hMPV F would be expected, and it is less likely to drive escape mutations focused on a single epitope. After prime and boost, RHMS-1 induced comparable levels of hMPV F/RSV F-specific serum IgG titers. Although the serum neutralization against RSV is not as potent as that against hMPV, RHMS-1 immunization completely protected the mice from both RSV and hMPV challenges, suggesting RHMS-1 is a promising antigen that can be used as a vaccine to induce cross-neutralizing and cross-protecting antibodies against RSV and hMPV. These findings also suggest additional optimization of antigen and adjuvants is needed to optimize the elicited neutralizing antibody responses.

Our ELISA screening data showed that the human subjects tested had pre-existing immunity against both RSV F and hMPV F. Interestingly, subjects had an overall higher frequency of RSV F-specific B cells than hMPV F-specific B cells, which is likely due to the higher prevalence of RSV than hMPV. Since initial exposures to an antigen can influence subsequent immune responses against similar antigens, termed original antigenic sin (69), pre-existing immunity to RSV or hMPV may affect the efficacy of RHMS-1 or other RSV and hMPV F-based vaccine candidates. To address this problem, future studies will be needed to test RHMS-1 in animal models that are pre-immune to RSV and hMPV. Furthermore, despite the encouraging results obtained from the RHMS-1 immunization/challenge in this study, it is still necessary to compare the efficacy of RHMS-1 versus a mixture of RSV F and hMPV F in animal models.

Previous studies have shown that mice immunized with either pre-fusion RSV F or post-fusion hMPV F did not induce significant cross‐neutralizing antibodies (29). Similar results were observed in this study with pre-fusion RSV F and monomeric hMPV F. The serum of 9 out of 16 mice immunized with RSV F + AddaS03 showed little binding to monomeric hMPV F just above the detection limit, while all of the monomeric hMPV F + AddaS03 immunized mice serum had moderate binding to RSV F. However, the serum of both groups failed to cross-neutralize the viruses *in vitro* (**Figures 6B–E**). Interestingly, the lung virus counts in RSV F immunized/hMPV challenged and hMPV F immunized/RSV challenged groups are reduced ~10 fold compared to mock immunized/hMPV challenged and mock immunized/RSV challenged groups, respectively (**Figure 6F**), indicating poorly and non-neutralizing, cross-reactive antibodies could play a role in limiting virus replication in the lungs of mice, possibly through Fc-mediated effector functions that need to be further characterized. Based on our current data, vaccine-induced immunopathology is not expected in animals treated with RHMS-1, because 1) dominant pre-fusion conformation particles of RHMS-1 were observed by negative-stain EM; 2) there is no significant change of Th2-associated cytokine levels in the post-challenge lungs from RHMS-1 vaccinated groups vs. the other control groups. Nevertheless, future studies in more permissive animal models such as cotton rats and African Green Monkeys will be needed to further evaluate the efficacy and safety of RHMS-1, as well as studies using a formalin-inactivated virus comparators to assess pathology. While not directly assessed in this report, it is not expected that RHMS-1 would cause liver toxicity due to a record of safety for RSV F and hMPV F in animal models and humans (70–73).

Although the RHMS-1 construct appears to be a pre-fusion trimer by negative-stain electron microscopy, the stability of this protein requires further evaluation, and could likely be optimized to improve both stability and antigenicity using computational protein design tools. For example, by using interprotomer disulfides (IP-DSs) that link protomers of the hMPV F trimer, both stabilized pre-fusion and post-fusion F proteins elicited significantly higher neutralizing responses than the hMPV F proteins without IP-DSs (74). Such a strategy could also be applied to stabilize the RHMS-1 construct. In addition, it would be interesting to further explore other vaccine platforms for RHMS-1. For example, mRNA vaccines can elicit robust antibody titers as well as T cell responses, which were not examined in this study. Live-attenuated vaccine would be another potential platform as it can induce mucosal immunity to prevent viral transmission, however, the fusogenicity of RHMS-1 will need to be assessed to determine if incorporation into live-attenuated vaccine platforms is warranted.

In summary, we generated and evaluated a chimeric RSV F and hMPV F protein, RHMS-1. To our knowledge, this is the first immunogen that elicits a protective immune response against both RSV and hMPV. Moreover, RHMS-1 can be readily applied to both traditional and novel vaccine delivery platforms like viral vectors, VLPs, nanoparticles, and mRNA. Further optimization of RHMS-1 could lead to a safe and effective universal Pneumovirus vaccine.

## Data availability statement

The original contributions presented in the study are included in the article/supplementary materials, further inquiries can be directed to the corresponding authors.

## Ethics statement

The studies involving human participants were reviewed and approved by University of Georgia IRB, and subjects were recruited to the University of Georgia Clinical and Translational Research Unit. The patients/participants provided their written informed consent to participate in this study. The animal study was reviewed and approved by University of Georgia IACUC committee.

## Author contributions

JH and RM conducted the experiments. JH, RM, and JJM. analyzed the data. JH and JJM wrote and revised the manuscript. RM revised the manuscript. JJM procured funding. All authors contributed to the article and approved the submitted version.

## Funding

These studies were supported by National Institutes of Health grants 1R01AI143865 (JJM) and 1K01OD026569 (JJM). The funders had no role in study design, data collection and analysis, the decision to publish, or preparation of the manuscript. Molecular graphics and analyses performed with UCSF ChimeraX, developed by the Resource for Biocomputing, Visualization, and Informatics at the University of California, San Francisco, with support from National Institutes of Health R01-GM129325 and the Office of Cyber Infrastructure and Computational Biology, National Institute of Allergy and Infectious Diseases.

## Conflict of interest

JH and JJM are listed as inventors on a provisional patent application describing the vaccine candidate.

The remaining author declarers that the research was conducted in the absence of any commercial or financial relationships that could be construed as a potential conflict of interest.

## Publisher’s note

All claims expressed in this article are solely those of the authors and do not necessarily represent those of their affiliated organizations, or those of the publisher, the editors and the reviewers. Any product that may be evaluated in this article, or claim that may be made by its manufacturer, is not guaranteed or endorsed by the publisher.
